# Description of a New Galapagos Giant Tortoise Species (*Chelonoidis*; Testudines: Testudinidae) from Cerro Fatal on Santa Cruz Island

**DOI:** 10.1371/journal.pone.0138779

**Published:** 2015-10-21

**Authors:** Nikos Poulakakis, Danielle L. Edwards, Ylenia Chiari, Ryan C. Garrick, Michael A. Russello, Edgar Benavides, Gregory J. Watkins-Colwell, Scott Glaberman, Washington Tapia, James P. Gibbs, Linda J. Cayot, Adalgisa Caccone

**Affiliations:** 1 Department of Biology, University of Crete, Vassilika Vouton, Gr-71300, Heraklion, Greece; 2 Natural History Museum of Crete, University of Crete, Knossos Av., GR-71409, Heraklion, Greece; 3 Department of Ecology and Evolutionary Biology, Yale University, 21 Sachem St. New Haven, Connecticut, 06520, United States of America; 4 Life and Environmental Sciences, University of California, Merced, 5200 N Lake Rd, Merced, California, 95343, United States of America; 5 Department of Biology, University of South Alabama, LSCB 123, 5871 USA Dr. N, Mobile, Alabama, 36688, United States of America; 6 Department of Biology, University of Mississippi, Oxford, Mississippi, 38677, United States of America; 7 Department of Biology, University of British Columbia, Okanagan Campus, Kelowna, BC V1V 1V7, Canada; 8 Division of Vertebrate Zoology, Yale Peabody Museum of Natural History, 170 Whitney Avenue, New Haven, Connecticut, 06520, United States of America; 9 Department of Applied Research, Galapagos National Park Service, Puerto Ayora, Galapagos, Ecuador; 10 Galapagos Conservancy, Fairfax, Virginia, 22030, United States of America; 11 College of Environmental Science & Forestry, State University of New York, Syracuse, New York, 13210, United States of America; Fordham University, UNITED STATES

## Abstract

The taxonomy of giant Galapagos tortoises (*Chelonoidis* spp.) is currently based primarily on morphological characters and island of origin. Over the last decade, compelling genetic evidence has accumulated for multiple independent evolutionary lineages, spurring the need for taxonomic revision. On the island of Santa Cruz there is currently a single named species, *C*. *porteri*. Recent genetic and morphological studies have shown that, within this taxon, there are two evolutionarily and spatially distinct lineages on the western and eastern sectors of the island, known as the Reserva and Cerro Fatal populations, respectively. Analyses of DNA from natural populations and museum specimens, including the type specimen for *C*. *porteri*, confirm the genetic distinctiveness of these two lineages and support elevation of the Cerro Fatal tortoises to the rank of species. In this paper, we identify DNA characters that define this new species, and infer evolutionary relationships relative to other species of Galapagos tortoises.

## Introduction

Giant Galapagos tortoises are icons of the Galapagos archipelago. They represent a classic example of an island adaptive radiation [1, 2], and are keystone herbivores [3]. Despite their prominence, the taxonomy of Galapagos tortoises has long been debated. Van Denburgh [4] originally recognized 14 species (13 of them named) within the genus *Testudo* based on island of origin and differences in carapace morphology. Since then, the taxonomy of the group has undergone recurring changes. First, Mertens and Wermuth [5] demoted described groups to the subspecies level, under the name *Testudo elephantopus*. Next, on the basis of morphological data, Loveridge and Williams [6] established *Geochelone* (Fitzinger, 1835) as the most appropriate genus for Galapagos (and many other) tortoises, and placed all the Galapagos forms in one species (*G*. *elephantopus*) within the subgenus *Chelonoidis* (Fitzinger, 1856; also containing mainland South American species). More recently, Bour [7] promoted *Chelonoidis* to generic status and elevated the subspecies to species. Despite a nomenclatural review by Pritchard [8] arguing for *Geochelone* and *Chelonoidis* as the appropriate genus and subgenus respectively, genetic data presented by Le, Raxworthy [9] indicated that *Geochelone* is polyphyletic and thus the generic status of *Chelonoidis* is supported.

Within his monograph, Van Denburgh [4] identified four general groups based on carapace shape: “saddle-back” (high anterior opening), “dome” (rounded cupola-like form), “intermediate” (between saddle-back and domed forms), and “unknown” (museum remains for which shape information is lacking). Tortoises from the islands of Española, San Cristóbal, Pinzón, Pinta, Floreana, Santa Fe (undescribed) and Fernandina are considered to be saddlebacks (the latter four taxa are now extinct). Tortoises from San Cristóbal Island, and Santiago Island have a carapace with an intermediate shape. Those from Isabela Island and Santa Cruz Island are domed [4, 10]. Saddleback tortoises have also been reported from northern Isabela, likely the result of human-mediated translocations [11]. Although useful for morphologically classifying tortoises, variation does exist within these three broadly defined carapace shapes [10].

Some authors have argued that Galapagos giant tortoise taxa should be considered subspecies [8], advocating for the synonymy of species described by Van Denburgh [4]; others accept the species status of all taxa except for four of the five named species on Isabela Island (the fifth being *C*. *becki*), which are lumped in a single species, *C*. *vicina* [12]. Genetic studies based broadly on mitochondrial DNA (mtDNA) sequence data support the evolutionary distinctiveness of described taxa [1, 2, 11, 13–16] with the clear exception of tortoises once found on Rábida Island, which are also likely human-mediated transplants [16]. Collectively, these studies further revealed that most populations from different islands represent clades and therefore independent evolutionary (and conservation) units. Moreover, geographically isolated populations within islands (e.g., those on separate volcanoes on Isabela Island) are readily distinguishable on the basis of nuclear microsatellite data, which indicates little or no gene flow among them [11, 15, 17–21].

**Fig 1:**
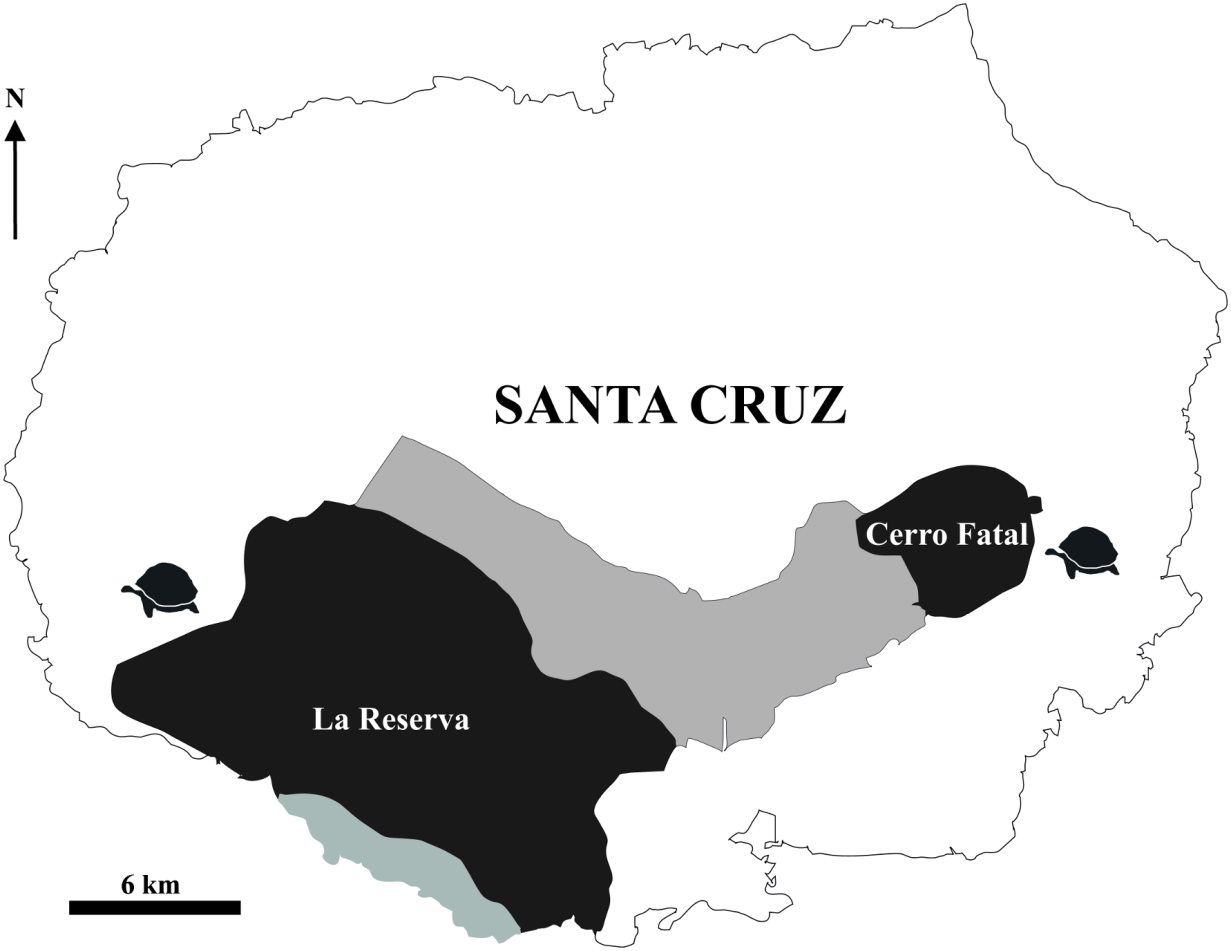
Geographic distribution of the two known lineages of giant tortoises on Santa Cruz Island: *Chelonoidis porteri* (Reserva) and *Chelonoidis* sp. nov. (Cerro Fatal) (indicated in dark gray). Light gray area connecting the distribution areas of the two species indicates agricultural land. Modified from Russello et al. [11].

Tortoises on Santa Cruz Island are currently considered members of a single named species, *C*. *porteri* (formerly Testudo porteri) [22] associated with the large population (“Reserva”) occurring on the island’s southwestern slopes in a mesic region of the island. This population occupies an area of ~156 km^2^ and includes 2,000–4,000 individuals [8, 23, 24]. A second tortoise population (“Cerro Fatal”) on the eastern side of Santa Cruz Island has long been recognized but considered a member of *C*. *porteri* (Fig 1). This population comprises vastly fewer tortoises (several hundred individuals) and occupies a smaller and dryer area (~40 km^2^) than the Reserva population from which it is separated by approximately 20 km (Fig 1) [25]. Although individuals of both populations exhibit a domed carapace morphology, morphological analyses indicated that tortoises from the two populations differ in size and shape [20, 26, 27]. Genetically, Reserva and Cerro Fatal tortoises are among the most divergent taxa within the archipelago: they belong to different major mtDNA clades [11, 16, 20] and were likely derived from separate colonizations of Santa Cruz Island from different source islands. Reserva tortoises are part of the oldest lineage in the archipelago (diverged ~1.74 million years ago, Mya), nested in a sub-clade including Isabela, Floreana and Pinzón Island tortoises. Cerro Fatal tortoises are much younger (~0.43 Mya), being most closely related to the tortoises from San Cristóbal, Pinta, and Española Islands [16]. Patterns and levels of genetic divergence based on nuclear microsatellite data support the relationships identified by mtDNA data. Each of the two taxa have numerous private alleles, implying very little recent gene flow, and they are as genetically divergent from each other as the other named species are from one another [11, 13, 20]. Previous studies have also revealed the existence of a limited amount of introgression between the two taxa [11, 13, 20], which is not unexpected given their geographical proximity. Over the last century, portions of the ranges of both species have been converted to farmland; the agricultural zone, a band stretching across the southern slope of the island from west to east, now provides a uniform habitat connection between the two species’ ranges. Moreover, the zone currently has many human residents, thus increasing the potential for human-mediated transport of tortoises.

Given the genetic distinctiveness of the Reserva and Cerro Fatal populations and the current application of a single name (*C*. *porteri*) to all Santa Cruz Island tortoises, we sought to clarify their taxonomy by integrating data from extant populations with those from museum specimens, including the *C*. *porteri* holotype (Rothschild 1903), and two from Cerro Fatal (Fig 1). We analyzed nuclear microsatellite and mtDNA genetic data from both sample sets to (1) confirm the genetic distinctiveness of the two tortoise populations, (2) clarify the genetic assignment of the holotype for *C*. *porteri*, (3) identify diagnostic genetic characters that define a new species from Cerro Fatal, and (4) determine the holotype for the new species.

## Material and Methods

### Museum specimens

Three specimens representing Santa Cruz Island tortoises were obtained from natural history museum collections: a skull from the University of Wisconsin Zoological Collection (UWZS; USW32700), collected in the Cerro Fatal area in 1991; an incomplete carapace section (CF_March2010) from the museum at the Charles Darwin Research Station in Puerto Ayora, Santa Cruz Island, collected in 2010 from Cerro Fatal; and the *C*. *porteri* holotype from the London Tring Museum (reg. no. BMNH 1949.1.4.38 or BMNH-1949; formerly Testudo porteri) [22] collected by R.H. Beck in 1902 with site information limited only to island of origin.

DNA extractions from museum specimens were performed in two physically isolated laboratories dedicated to the extraction of ancient DNA (aDNA): at Yale University and the University of Crete (Greece). All standard precautions were followed to prevent contamination by extant specimens. Detailed descriptions of the methods used to extract, amplify, and sequence DNA from the bones of the giant Galapagos tortoises are provided in the S1 File. Approximately 700 bp of the mtDNA control region (CR) and 12 microsatellite loci were amplified from all museum specimens using previously published primers and protocols [11, 16, 18, 28].

### Genetic analyses

To investigate evolutionary relationships of the three museum samples in the context of all available data from extant and extinct giant Galapagos tortoise species, we combined the new mtDNA sequences with 123 unique CR haplotypes from tortoises of all the named extinct and extant species identified by previously published mtDNA studies [1, 2, 11, 13–16, 18, 20] and three outgroup taxa from continental South America (*C*. *chilensis*, *C*. *denticulata*, and *C*. *carbonaria*) [1, 2]. Control region sequences were aligned in MAFFT v.7 [29] using default settings. Bayesian Inference (BI) phylogenetic analysis was conducted in MrBAYES v.3.2.1 [30]. The TrN + G model of nucleotide substitution was used, selected according to the Bayesian Information Criterion (BIC) implemented in jModelTest v. 2.1.1 [31], ignoring the models that include both gamma distribution and invariable sites [32]. Bayesian Inference phylogenetic analysis was run four times (independent random starting trees) with eight chains for each run of 10^7^ Markov chain Monte Carlo (MCMC) generations, sampling from the chain every 100^th^ generation. This generated an output of 10^5^ trees. To confirm that the chains had achieved stationarity, we evaluated “burn-in” by plotting–*ln*L tree scores and tree lengths against generation number using Tracer v.1.5.0 [33]. The–*ln*L tree scores stabilized after approximately 2×10^6^ generations and the first 25% of trees were discarded as a conservative measure to avoid the possibility of including stochastically generated, sub-optimal trees. A majority-rule consensus tree was then derived from the posterior distribution of trees, with posterior probabilities calculated as the percentage of samples that recovered any particular node. We also ran the analysis with no data to sample the prior distributions for each parameter to confirm that the priors were not driving the outcomes.

To estimate levels of genetic diversity within each of the two tortoise populations, 70 mtDNA sequences of *C*. *porteri* from Reserva and 51 from Cerro Fatal tortoises from previous studies were combined with the sequences collected from museum specimens in this study, creating a dataset of 124 mtDNA control region sequences. The number of segregating sites (*S*) and haplotype (*H*_*D*_) and nucleotide (*π*) diversity were computed using DnaSP v. 5.10 [34]. A haplotype network was generated using statistical parsimony [35] implemented in TCS v.1.13 with the 95% confidence criterion enforced [36].

Genotypic data from 12 nuclear microsatellite loci were used to further investigate genetic distinctiveness of the two populations. Our reference database included genotypic data from extant samples collected for previous studies from Santa Cruz Island (Cerro Fatal; n = 21, Reserva; n = 34; [11, 13, 17, 20]) and the three museum samples analyzed in this study. Given that null alleles, stuttering signals or large allelic dropouts could contribute to ‘false positive’ homozygous patterns, the pure Cerro Fatal and La Reserva populations were examined using MICROCHECKER v2.2.3 [37] with no evidences for scoring error due to stuttering and large allelic dropouts or null alleles. To assign the museum samples to a particular taxon, we used the Bayesian clustering method implemented in STRUCTURE v2.3 [38]. Membership coefficients (*Q*-values) from individuals collected in either Cerro Fatal or Reserva were used to assign individuals to a particular population of origin following a MCMC simulation of 10^8^ steps after an initial ‘burnin’ of 10^7^ steps. The MCMC sampling frequency was set at default. Analyses were run using an admixture model using locality origin as prior information for cluster assignment of extant samples, but not for the museum samples in order to be assigned to one of the two populations. The analysis was repeated 20 times to assess consistency of results. CLUMPP [39] was used to combine and summarize parameter estimates from STRUCTURE, with input files prepared using STRUCTURE HARVESTER [40]. Results were then plotted using DISTRUCT [41].

GENECLASS2 v2.0 [42] was also used to identify migrant individuals, individuals with mixed ancestry, and individuals that do not strongly assign to any population. To compute the probability of each individual’s belonging to a set of reference populations, assignment tests were performed using direct and simulation approaches based on the partial Bayesian method of Rannala and Mountain [43] and by setting the threshold for exclusion of individuals to 0.05.

Average allelic richness (corrected for sample size by rarefaction) per locality was calculated in the HIERFSTAT package [44] for R (http://www.R-project.org/). Observed and expected heterozygosity were calculated using Arlequin v3.5.1.3 [45]. Weir and Cockerham’s [46] estimate of *F*_*IS*_ (inbreeding coefficient) was calculated using GenePop v4.0.10 [47].

To assess levels of genetic differentiation between the Reserva and Cerro Fatal tortoises, we compared the mtDNA and microsatellite distances between these two populations with those found between other named species of Galapagos tortoises. For these analyses, we excluded introgressed individuals (i.e. used only purebred individuals) as we were interested in estimating the amount of evolutionary divergence between the two taxa. For mtDNA sequence data, we calculated divergences using two metrics: uncorrected *p*-distance, and maximum likelihood-corrected distances (calculated in PAUP* v4.0b10) [48]. For the mtDNA-based metrics, non-redundant haplotypes were the units of analysis (123 haplotypes from previous studies plus one from this study; see results below), excluding the haplotypes from the individuals that showed signs of introgression (n = 10), yielding 6441 interspecific pairwise comparisons. Similarly, for microsatellite data, we used two metrics that, in combination, can be informative about whether divergences occurred on recent *vs*. older timescales (i.e., *F*_ST_ *vs*. *R*_ST_ calculated in GENEPOP and R_ST_CALC v2.2 [49], respectively). For the microsatellite-based metrics, populations were the unit of analysis (i.e., 79 interspecific pairwise comparisons). In addition to Cerro Fatal and Reserva lineages, the taxa for which all possible pairwise comparisons were performed were *C*. *hoodensis*, *C*. *chathamensis*, *C*. *abingdoni*, *C*. *ephippium*, *C*. *darwini*, *C*. *vandenburghi*, *C*. *microphyes*, *C*. *guntheri*, *C*. *vicina*, *C*. *elephantopus*, and *C*. *becki*.

### Nomenclatural Acts

The electronic edition of this article conforms to the requirements of the amended International Code of Zoological Nomenclature, and hence the new names contained herein are available under that Code from the electronic edition of this article. This published work and the nomenclatural acts it contains have been registered in ZooBank, the online registration system for the ICZN. The ZooBank LSIDs (Life Science Identifiers) can be resolved and the associated information viewed through any standard web browser by appending the LSID to the prefix "http://zoobank.org/". The LSID for this publication is: urn:lsid:zoobank.org:pub:065FBB00-835F-421E-860A-D06C15465D1E. The electronic edition of this work was published in a journal with an ISSN, and has been archived and is available from the following digital repositories: PubMed Central, LOCKSS.

## Results

### Phylogenetic placement of tortoises from Reserva and Cerro Fatal

We sequenced the mtDNA control region from the three museum specimens, with sequence lengths varying from 390 bp (CF_March2010, GenBank accession number: KT192435) to 697 bp (USW32700 and BMNH-1949) (GenBank accession numbers: KT192434 and KT192436, respectively). All three mtDNA sequences represented a different haplotype. The haplotypes belonging to samples USW32700 and CF_March2010 were identical to published haplotypes found only in Cerro Fatal animals (CF2; AY956612 and CF1; AY097977, respectively). The third haplotype carried by the *C*. *porteri* holotype (BMNH-1949) was novel (increasing the total number of known unique haplotypes across all extinct and extant Galapagos tortoises to 124), but only 3–4 mutational steps away from the four previously detected haplotypes found only in the extant tortoise lineage from Cerro Fatal.

**Fig 2:**
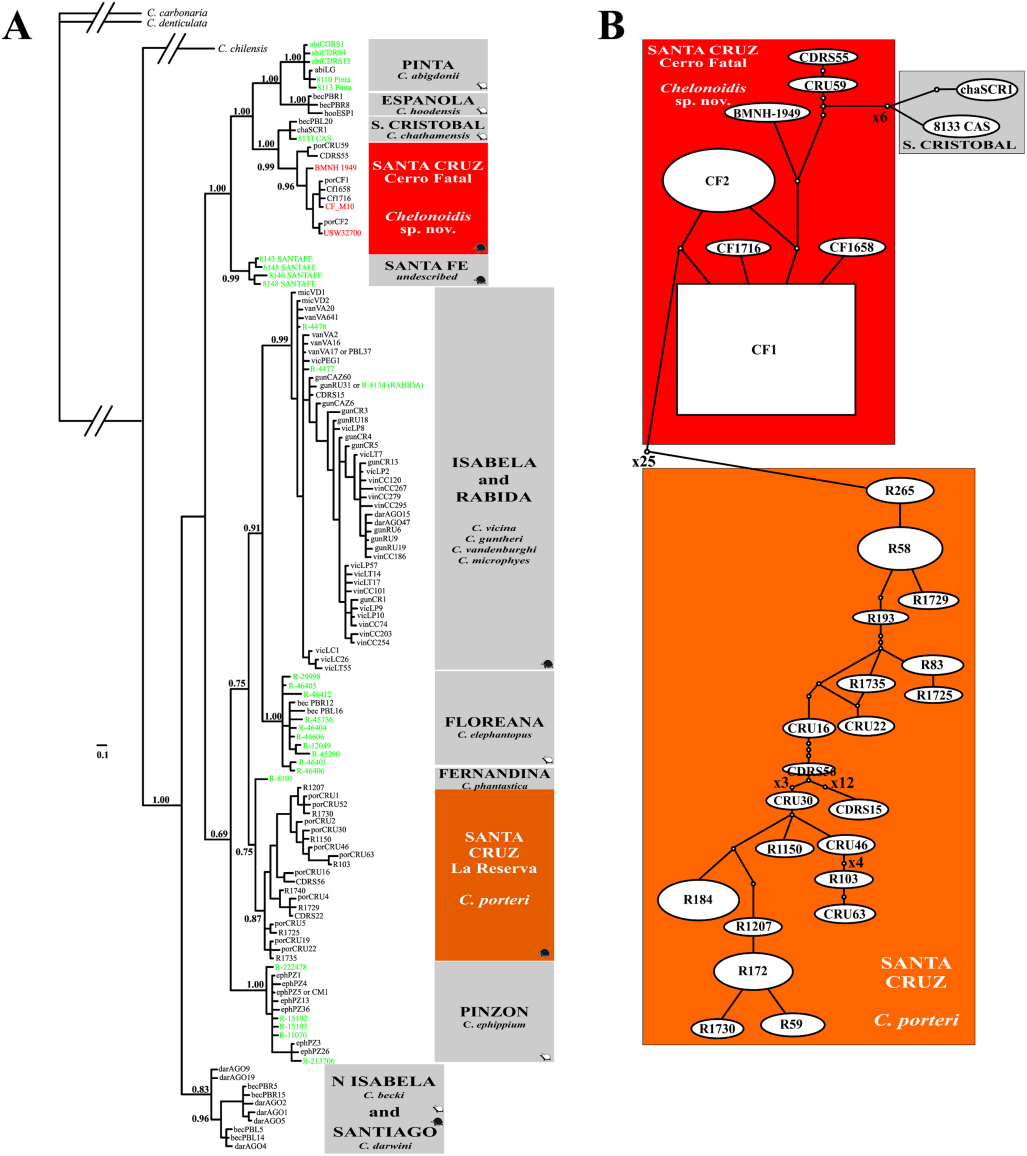
**(A)** Bayesian Inference (BI) tree reconstructed from the dataset including all unique mtDNA control region haplotypes previously sampled from extant and extinct species as well as the three museum specimens of giant Galapagos tortoises analyzed in this study. Numbers on branches indicate posterior probabilities. Only the nodal support values for the major lineages are presented. Red and green colors identify museum samples analyzed in the present and previous studies, respectively. (**B)** Haplotype network showing matrilineal diversity recovered from 70 sequences of *C*. *porteri* from Reserva, 51 sequences of the lineage from Cerro Fatal, and 2 sequences of *C*. *chathamensis* from San Cristóbal Island. Twenty-five inferred mutations separate the haplogroups of *Chelonoidis* sp. nov. from Cerro Fatal from the ones from *C*. *porteri* from Reserva.

Bayesian Inference phylogenetic analysis including all previously detected mtDNA control region haplotypes for extant and extinct Galapagos tortoises (n = 123), the museum specimens sequenced here (n = 3), and outgroup taxa (n = 3) revealed that the Santa Cruz Island lineages are paraphyletic (arithmetic mean: *ln*L = -3903.81) (Fig 2A), as reported previously [11, 15, 16]. All of the museum specimens sequenced here, including the *C*. *porteri* holotype, cluster within the mtDNA lineage of Cerro Fatal with high node support (posterior probability = 0.99; Fig 2A). The lineage from Cerro Fatal is sister to the species from San Cristóbal Island (*C*. *chathamensis*), and both are included in a clade with species from the islands of Pinta (*C*. *abingdoni*), Española (*C*. *hoodensis*), and Santa Fe (undescribed). In contrast, the lineage from Reserva (*C*. *porteri*) falls within a clade with species from Isabela (*C*. *becki*), Floreana (*C*. *elephantopus*), Fernandina (*C*. *phantastica*), and Pinzón (*C*. *ephippium*) Islands.

### MtDNA diversity in Santa Cruz populations

Twenty-one haplotypes were recognized among the 70 control region sequences obtained from members of the Reserva lineage, while only seven haplotypes were detected among the 54 control region sequences from the Cerro Fatal population (Table 1). Compared to Cerro Fatal, tortoises from Reserva also exhibited substantially higher haplotypic diversity, nucleotide diversity, and number of segregating sites (Table 1). This result mirrors previous work on the two Santa Cruz taxa, which showed that they experienced very different demographic histories [11, 13, 14].

**Table 1:** Summary statistics from microsatellite (*N—F*_*IS*_) and mtDNA (*H*_*D*_—No of Haplotypes) data for the Cerro Fatal and Reserva populations of giant tortoises on Santa Cruz Island.

Population	N	*A*_*R*_	*H*_*E*_	*H*_*O*_	*F*_*IS*_	*H*_*D*_	*π*	*S*	*No* of haplotypes
Cerro Fatal	21	4.75	0.58	0.62	-0.10	0.57	0.003	9	7
Reserva	34	11.13	0.79	0.76	0.04	0.92	0.005	30	21

*N*: sample size; *A*_*R*_: rarefied allelic richness; *H*_*E*_: expected heterozygosity; *H*_*O*_: observed heterozygosity; *F*_*IS*_: inbreeding coefficient; *H*_*D*_: haplotype diversity; *π*: nucleotide diversity; *S*: number of segregating sites.

**Fig 3:**
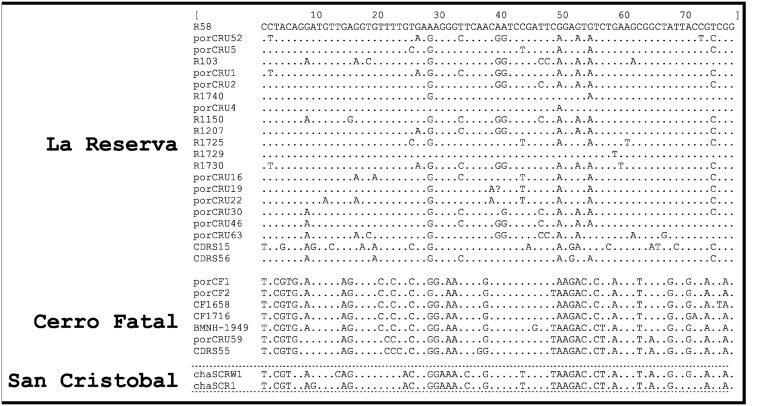
Polymorphic sites between *Chelonoidis* sp. nov. (Cerro Fatal—Santa Cruz), *C*. *chathamensis* (San Cristóbal), and *C*. *porteri* (Reserva—Santa Cruz). The position of diagnostic locations is relative to the Genbank record AY956622 for porCF1 from Cerro Fatal. - = gap position and K = G/T polymorphism.

The mtDNA haplotype network revealed two distinct haplogroups (Fig 2B): one contained the 21 haplotypes from Reserva, and the other consisted of seven haplotypes, four of which were retrieved from tortoises from Cerro Fatal (pure and unique CF haplotypes not otherwise detected across the archipelago; CF1, CF2, CF1658, and CF1716), two from Reserva (CRU59, CDRS55), and the *C*. *porteri* holotype (BMNH-1949). This second haplogroup is genetically closer to the one found in the San Cristóbal tortoises than the one in Reserva (6 *vs*. 25 mutational steps), confirming the sister relationship of the San Cristóbal and Cerro Fatal taxa (Figs 2A and 3). Furthermore, Cerro Fatal tortoises are diagnosably distinct from Reserva and *C*. *chathamensis*, its sister taxon from San Cristóbal Island (Fig 2A), as evidenced by 18 and six diagnostic mtDNA sites, respectively (Table 2).

**Table 2:** Diagnostic sites distinguishing *Chelonoidis* sp. nov. (Cerro Fatal—Santa Cruz), *C*. *chathamensis* (San Cristóbal) and *C*. *porteri* (Reserva—Santa Cruz). The position of diagnostic locations is relative to the GenBank record AY956622 for porCF1 from Cerro Fatal. Capital letters (nucleotides): diagnostic sites between *Chelonoidis* sp. nov. (Cerro Fatal) and *C*. *porteri* (Reserva); small letters (nucleotides): diagnostic sites between *Chelonoidis* sp. nov. (Cerro Fatal) and *C*. *chathamensis* (San Cristóbal); bold capital letters (nucleotides): diagnostic sites between *Chelonoidis* sp. nov. (Cerro Fatal) and both *C*. *porteri* (Reserva) and *C*. *chathamensis* (San Cristóbal). K = G/T, R = A/G, Y = C/T polymorphisms.

Species	Position of diagnostic sites
	011222222223333344566
	901133666780345817025
	750248037895376206552
*Chelonoidis* sp. nov.	CT**G**A**C**ggAA**T**GcACCATGGAA
*C*. *porteri*	TC**A**G**T**ggGG**C**AyGTTKCAAGG
*C*. *chathamensis*	CT**A**A**T**aaAA**C**GtACCATGRAA

Overall, the mtDNA sequence divergence between Reserva and Cerro Fatal is much higher (more than double in some cases) than corresponding values between each of these populations and named species from different islands (Table 3).

**Table 3:** Sequence divergences (%, uncorrected p-distance) among the mtDNA control region sequences of *Chelonoidis* sp. nov. (Cerro Fatal) and all the recognized species of giant Galapagos tortoises.

Species	1	2	3	4	5	6	7	8	9	10	11	12	13	14	15
1. *Chelonoidis* sp. nov. (Cerro Fatal)															
2. *C*. *porteri* (Reserva)	**4.5**														
3. *C*. *abingdoni*	3.4	5.4													
4. *C*. sp. (Santa Fe) (Undescribed)	3.1	3.9	2.6												
5. *C*. *hoodensis*	3.6	5.6	2.2	3.8											
6. *C*. *chathamensis*	1.4	4.8	2.7	2.7	3.2										
7. *C*. *darwini*	4.1	4.0	4.9	3.8	4.2	3.8									
8. *C*. *ephippium*	5.0	3.3	5.5	4.0	5.6	4.7	4.5								
9. *C*. *elephantopus*	4.1	2.2	4.9	3.7	5.4	4.7	3.9	3.4							
10. *C*. *guntheri*	4.5	2.8	4.7	4.1	4.9	4.6	4.2	3.9	2.2						
11. *C*. *microphyes*	4.4	2.3	4.6	3.5	5.1	4.8	4.1	3.5	1.9	1.0					
12. *C*. *vanderburghi*	4.5	2.5	4.6	3.6	5.2	4.7	4.3	3.7	2.1	1.0	0.3				
13. *C*. *vicina*	4.7	2.8	4.9	4.3	5.2	4.8	4.3	4.0	2.3	0.8	1.0	1.0			
14. *C*. *becki*	4.0	4.1	4.9	3.8	4.1	3.9	0.8	4.5	3.9	4.3	4.2	4.4	4.4		
15. Rábida’s lineage^#^	4.5	2.8	4.4	3.5	5.2	4.5	4.4	3.9	2.3	0.9	0.6	0.5	1.2	4.5	
16. *C*. *phantastica*	4.3	1.2	4.8	3.6	4.8	4.3	3.3	2.6	1.6	1.9	1.5	1.7	2.0	3.3	2.0

^#^ The haplotype of the lineage from the island of Rabida is nested within haplotypes found in extant species from the island of Isabela [16], supporting an early speculation that the Rabida tortoise did not belong to a distinct species, but rather to tortoises collected elsewhere and consumed by sailors on Rabida [8].

### Microsatellite genotypic diversity in Santa Cruz populations

Levels of microsatellite diversity are lower in Cerro Fatal than La Reserva, echoing the results of the mtDNA data in suggesting independent demographic histories and corroborating previous work [11, 13, 14].

**Fig 4:**
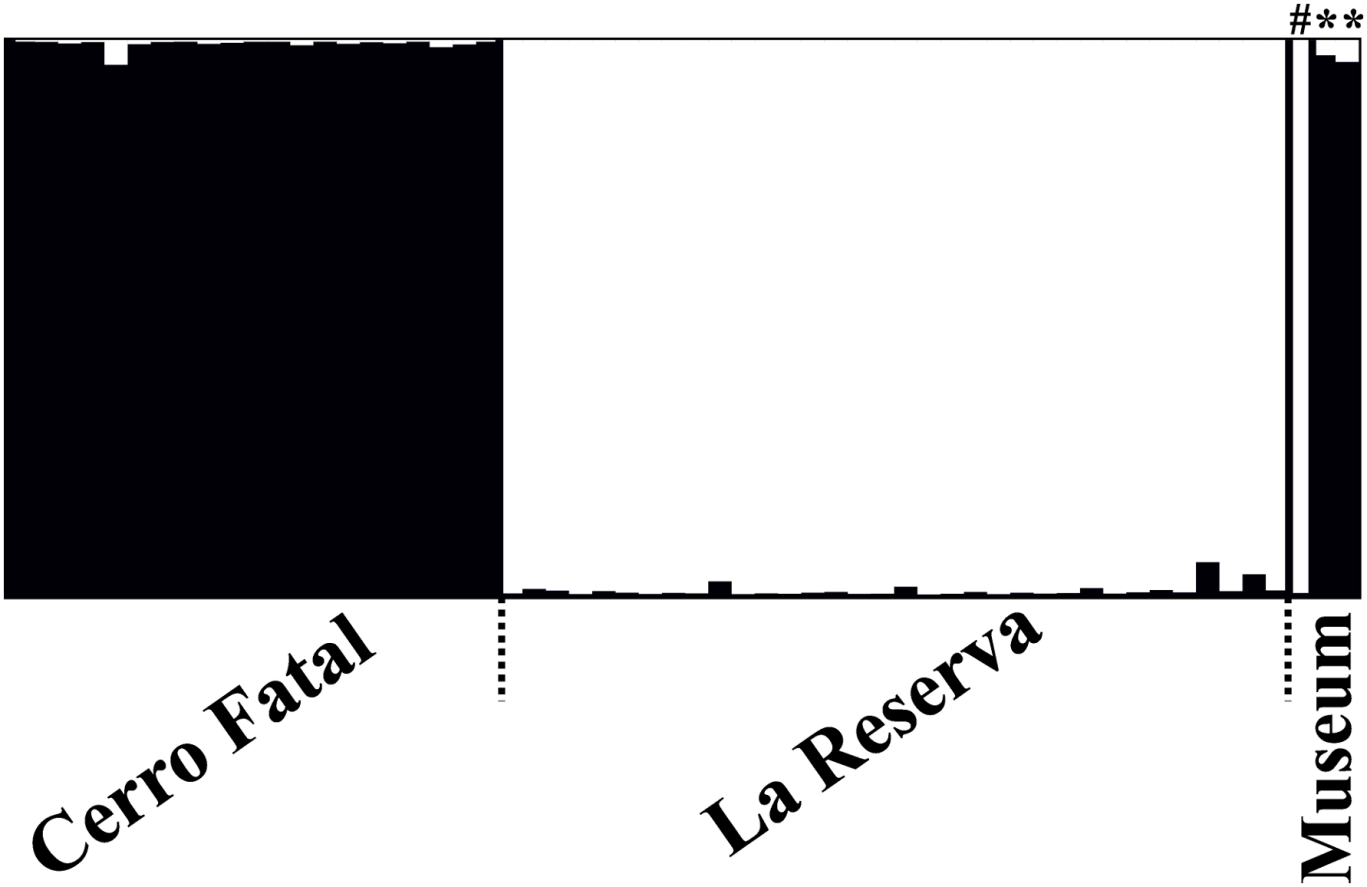
Genetic membership from Bayesian assignment tests in STRUCTURE for the three museum individuals, relative to the genotypic database representing the Cerro Fatal (black) and Reserva (white) giant tortoise populations. Each bar represents an individual and the proportional color of each bar represents the percentage membership (i.e., *Q-*value) in each of the reference clusters. Museum individuals include the *C*. *porteri* holotype (#) and the two Cerro Fatal specimens tested as putative candidates for the *Chelonoidis* sp. nov. holotype (*).

Bayesian clustering of genotypic data from 12 microsatellite loci revealed that the Cerro Fatal and Reserva populations constitute genetically distinct clusters relative to each other as well as to all other named Galapagos tortoises species (Fig 4) as has been reported previously [11, 13, 20]. The two museum specimens from Cerro Fatal (USW32700 and CF_March2010) cluster with the extant Cerro Fatal tortoises with high membership coefficients (*Q* = >96%), consistent with results from mtDNA data. The *C*. *porteri* holotype (BMNH-1949) groups with high membership (*Q* = 99%) with the extant tortoises from Reserva despite possessing a mtDNA haplotype more closely related to Cerro Fatal. The same results were obtained using GENECLASS: the USW32700 and CF_March2010 specimens have a very high probability of assigning to the Cerro Fatal group, while the BMNH-1949 is assigned with 100% probability to the La Reserva one (Table 4). Similar to results based on mtDNA, microsatellite genotypic diversity is substantially higher in Reserva tortoises compared to those from Cerro Fatal (Table 1). Further, tortoises from Cerro Fatal are characterized by significantly different allele frequencies at the 12 microsatellite loci and have three private alleles when compared to tortoises from La Reserva (GAL50: 171bp, GAL100: 104bp, and GAL159: 110bp).

**Table 4:** Museum specimens assignment according to the microsatellite genotypic data and the test of GeneClass2 are indicated by their percent assignment (%) and corresponding likelihood values (‘L1’ and ‘L2’).

Individuals	Pop	%	L1 [log(L)]	Pop	%	L2 [-log(L)]
BMNH 1949.1.4.38	La Reserva	100	33.328	Cerro Fatal	0.000	45.619
UWZS32700	Cerro Fatal	100	22.49	La Reserva	0.000	30.198
CF_March2010	Cerro Fatal	99.999	22.806	La Reserva	0.001	27.676

## Discussion

### More than one species

Island of origin and morphology have been useful in diagnosing Galapagos tortoise species [4], yet cases where taxa occupy the same island and have similar phenotypes (e.g., Isabela and Santa Cruz Islands) [1, 11, 13, 17] have resulted in unresolved taxonomic issues. This is the case with the tortoises of Santa Cruz, where two populations are currently described as a single species because they live on the same island (albeit with distinct distribution ranges and nesting areas) and members of both possess a domed carapace. Chiari et al. [20] and Chiari & Claude [26] summarized evidence for the existence of two evolutionary lineages on Santa Cruz, which includes being distinct reciprocally monophyletic groups within the Galapagos tortoise radiation [1, 2, 11, 15, 16, 50], having differences in nuclear microsatellites ([20] and this work), and statistically distinct morphology [20, 26, 27].

**Fig 5:**
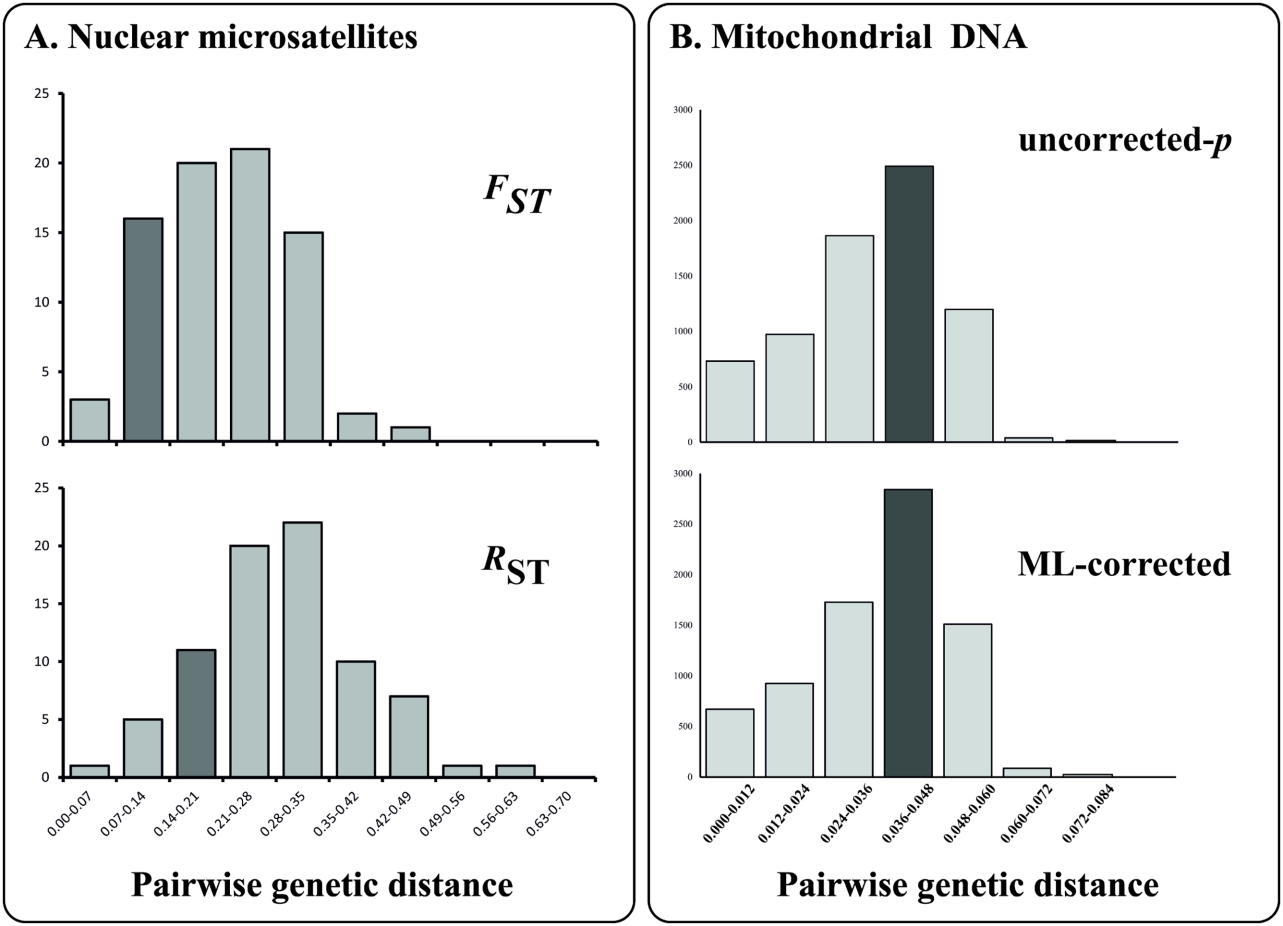
Frequency distribution of genetic distances between different Galapagos giant tortoise species (A: microsatellite DNA, B: mitochondrial DNA). For each histogram, a dark gray column indicates where the observed genetic distance between the Cerro Fatal and Reserva tortoises falls. Left: Microsatellite genetic distances calculated from purebred individuals in the reference measured using *F*_ST_ (top) or *R*_ST_ (below). Right: DNA sequence genetic distances based on mtDNA haplotypes from purebred individuals in the reference database, measured using uncorrected p-distances (top), or maximum likelihood (ML)-corrected distances (below).

Both mtDNA and microsatellite genetic distances between the two Santa Cruz populations are similar to, if not greater than, genetic distances observed between previously described and well-accepted species of giant tortoises inhabiting other islands in the Galapagos (Table 3 and Fig 5) [1, 11, 17, 51]. Although using genetic distance values for species delimitation is not a practice we ascribe to because of its several flaws [51], the fact that the two Santa Cruz taxa are as divergent for both types of genetic markers as other named Giant Galapagos tortoise species reinforces the argument that they should be given the same taxonomic rank as the others.

Also shown by previous studies [13, 20, 52], the mtDNA data revealed that a small proportion of individuals (~3%) are of mixed origin between the two populations. As argued elsewhere, we suggest that this is due to translocation and consequent hybridization of a very small set of individuals [13, 52]. These rare introgressive events have also been reported for other named species of Galapagos tortoises, where they have been interpreted as the result of human or environmentally driven secondary contacts among recently diverged species rather than natural oceanic dispersal or ancestral polymorphisms [1, 2, 15, 18, 21, 52].

### Holotype identification

In order to define the holotype for the new species from Cerro Fatal on Santa Cruz Island, we first checked the genetic assignment of the *C*. *porteri* holotype (reg. no. BMNH 1949.1.4.38). This specimen has nuclear genetic assignment to the lineage from Reserva (Fig 4) and a mtDNA haplotype that clustered with those endemic to Cerro Fatal (Fig 2). Thus, this specimen belongs to the rare cohort of hybrid individuals between the two lineages identified among the living tortoises sampled [20]. Therefore, the holotype of *C*. *porteri* relates to more than one taxon. Based on the International Code of Zoological Nomenclature (Article 17), “the availability of a name is not affected even if it is found that the original description or name-bearing type specimen(s) relates to more than one taxon, or to parts of animals belonging to more than one taxon (17.1) or it is applied to a taxon known, or later found, to be of hybrid origin (17.2).” Thus, although our genetic data clearly identify the hybrid origin of the *C*. *porteri* holotype, this species name is still valid. We further suggest that this name should be associated with the Reserva population, given that the Cerro Fatal population was assessed taxonomically only recently, and thus, it is unlikely that BMNH 1949.1.4.38 was sampled in that area. The finding that the nuclear DNA of this specimen strongly assigns it within the Reserva lineage further supports this possibility, as this tortoise is likely to have been the result of a remote introgression event followed by multiple mating events involving only Reserva tortoises.

Based on the results of the phylogenetic and population genetic analyses, we selected the skull from UWZS32700 as the holotype for the new species, *Chelonoidis* sp. nov., from Cerro Fatal in Santa Cruz.

**Fig 6:**
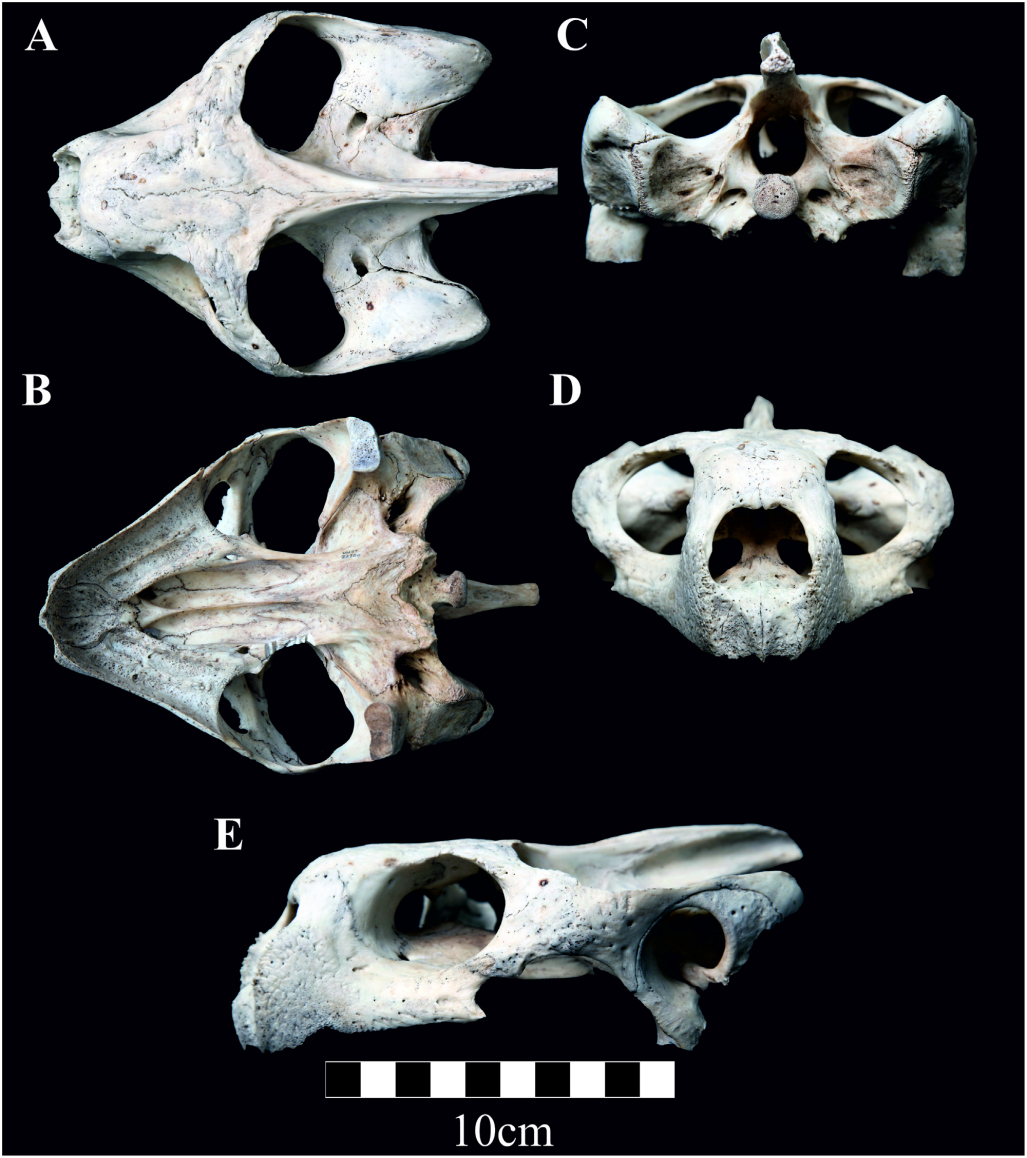
A-E. The skull of the museum specimen UWZS 32700, holotype for *Chelonoidis* sp. nov. from Cerro Fatal in Santa Cruz (A: dorsal, B: ventral, C: occipital, D: frontal and E: lateral view).

**SYSTEMATICS**
**Class REPTLIA Laurenti, 1768**
**Order TESTUDINES Linnaeus, 1758**
**Family TESTUDINIDAE Batsch, 1788**
**Genus *Chelonoidis* Fitzinger, 1835**
***Chelonoidis donfaustoi* sp. nov. Poulakakis, Edwards, and Caccone**
**urn:lsid:zoobank.org:act:333D161C-2BA0-43D3-B84E-B0FBC859787D**
**(Fig 6A–6E)**

Common name: Eastern Santa Cruz Tortoise. To distinguish the two extant lineages now recognized on Santa Cruz Island, we also propose to substitute for the current common name *Chelonoidis porteri* from “Santa Cruz tortoise,” which inappropriately subsumes both lineages, to the “Western Santa Cruz tortoise” to more clearly distinguish these two, distinct taxa.

### Holotype

Below we provide the morphological description of the skull from the designated *C*. *donfaustoi* holotype. This morphological description is not intended to diagnose the new species, but to clearly describe the specimen. Anatomical terms follow Gaffney (1979). Specimen UWZS 32700 is a skull (Fig 6A–6E) and single carapace marginal scute. The skull is nearly equal in basicranial length and maximum width (Table 5). The quadratojugal broadly contacts the jugal anteriorly and narrowly contacts the postorbital dorsally. Anterior edge of maxilla is rough. Posterior edge of maxilla does not extend past the contact of maxilla to jugal. In lateral view (Fig 6E) the squamosal is only narrowly visible dorsal to the quadrate and is not visible posterior to the quadrate. Dorsally the squamosal only narrowly contacts the opisthotic (Fig 6A). Contact of the pre-frontals to the frontals is broad and V-shaped, with the pre-frontal extending posteriorly forming the majority of the medial margin of the eye socket. The parietal extends nearly as far dorsally as the pre-frontal extends posteriorly and bisects the frontals. Maxilla contact with pre-frontal extends dorsally and is visible when the skull is viewed dorsally. Preootic is wider than long and does not extend posteriorly much beyond the foramen stepedio-temporale. Supraoccipital extends posteriorly well beyond the squamosal. Contact between basisphenoid and basioccipital is V-shaped with the lateral contact extending posteriorly. Vomer does not contact basisphenoid. Palatine bone much longer than wide. Prefrontal visible ventrally making contact with the palatine and vomer.

**Table 5:** Morphometric data for UWZS 32700 compared to data presented in Crumly [53]. Sixteen measurements were taken from the skull of UWZS 32700 following those described by Crumly [53]. Measurements were taken to the nearest 0.01 mm using a Mitutoyo digital caliper.

Variable	Species					
	UWZS 32700	*ephippium*	*guntheri*	*porteri*	*vicina*	*chathamensis*
B	115.0	96.7	128.0	121.5	109.0	98.1
WAT	117.6	73.9	106.6	98.4	86.0	80.4
WO	40.2	25.1	35.4	37.0	28.4	28.4
HN	20.2	12.5	18.6	18.6	16.1	13.9
WN	24.5	17.0	25.1	23.1	21.3	18.5
LB	17.4	13.3	18.7	14.7	18.1	14.7
WB	19.2	14.6	19.1	17.1	15.8	13.8
WZ	18.6	9.3	14.3	13.3	12.6	10.1
WP	10.6	7.0	9.5	9.0	8.8	7.3
WS	12.9	7.3	12.0	9.5	9.6	7.9
DPV	3.2	3.2	4.2	4.2	3.7	3.1
LP	16.1	14.1	21.0	18.1	14.8	15.2
WFS	17.4	10.0	16.0	12.8	8.9	12.5
PW	30.5	19.2	25.9	26.1	21.9	19.0
APW	20.0	10.5	15.2	14.1	11.8	10.5
PC	11.2	8.6	10.6	13.3	8.5	8.2

B: Basicranial length; WAT: Width of skull at anterior tympanic opening; WO: Width between orbits; HN: Height of external narial opening; WN: Width of external narial opening; LB: Length of basisphenoid; WB: Width of basisphenoid; WZ: Width of quadratojugal; WP: Width of postorbital; WS: Width of jugal; DPV: Distance (greatest) from prepalatine foramina to vomer; LP: Length of preootic; WFS: Width of prootic at stapedial foramen; PW: Width of pterygoid waist; APW: Width of anterior premaxillae; PC: Length of sagittal contact of prefrontals.

#### Diagnosis

The new species can be diagnosed by a combination of genetic, morphological, and geographic distribution evidence.

#### Genetic characters

This species can be distinguished from all other Galapagos tortoise species by allele frequency differences at 12 microsatellite loci, which allow assignment of Cerro Fatal tortoises to their own genetically distinct cluster. This cluster is as genetically divergent from the other named species as the genetic clusters grouping them. Furthermore, a set of polymorphisms in the mitochondrial control region sequence (haplotypes) is unique to the Cerro Fatal taxon (Fig 3). In particular, all individuals from Cerro Fatal share a set of nucleotides that separates them from the *C*. *porteri* (Reserva) species on the same island and from *C*. *chathamensis* (San Cristóbal), the sister taxon to the Cerro Fatal tortoises.

Morphological characters: Although there are no diagnostic morphological characters that uniquely define the new species, linear and geometric morphometric analyses reveal consistent differences in mean shell size and shape between tortoises from Reserva and Cerro Fatal. Tortoises from Reserva are larger in size and have a relatively larger carapace with higher anterior opening than the Cerro Fatal tortoises [20, 26, 27].

Distribution: The new species is only found on the eastern side of Santa Cruz Island occupying an area currently estimated at about 40 km^2^ (Fig 1).

#### Etymology

The new species is named in honor of Fausto Llerena Sánchez who devoted 43 years of service (1971–2014) to giant tortoise conservation as a park ranger within the Galapagos National Park Directorate. “Don Fausto” was the primary caretaker of endangered tortoises in captivity, one of the first to explore tortoise habitat throughout the archipelago, and was well known for his work ethic, commitment to tortoise conservation, and collegiality. Several tortoise lineages in Galapagos remain extant in large part due to Don Fausto’s dedication, ingenuity, and patience.

## Conclusion

Genetic and morphological data confirm the existence of two tortoise species on Santa Cruz Island. We describe the tortoises from Cerro Fatal as a new species, *C*. *donfaustoi*. The recognition of *C*. *donfaustoi* as a new species has important conservation implications for both taxa. The revised taxonomy reduces the range of *C*. *porteri*, with a population of several thousand individuals, to occupying only the western and southwestern parts of Santa Cruz Island. It also confines *C*. *donfaustoi* to the eastern part of Santa Cruz Island, with a much smaller population size estimated currently at ca. 250 individuals.

From a conservation standpoint, recognition of this new species will help promote efforts to protect and restore it, given that its low abundance, small geographic range, and reduced genetic diversity make it vulnerable. In particular, further investigation is needed to better determine *C*. *donfaustoi*‘s population size and structure, range, movement patterns, location of nesting zones, and habitat requirements, as well as ongoing threats and effective ways to mitigate them. In an age of increasing human occupation of much of the higher elevations on Santa Cruz Island, maintaining the two species’ biological isolation is critical. Of particular importance is ensuring that no human-mediated transport of tortoises occurs between the two sides of Santa Cruz Island given that the two species’ ranges are now linked via a single agricultural zone.

## Supporting Information

S1 File Detailed descriptions of the methods used to extract, amplify, and sequence DNA from the bones of the giant Galapagos tortoises. (DOCX)
