# Supplementary material for: Description of a New Galapagos Giant Tortoise Species (Chelonoidis; Testudines: Testudinidae) from Cerro Fatal on Santa Cruz Island
Source: PLoS One. 2015 Oct 21;10(10):e0138779. doi: 10.1371/journal.pone.0138779 (PMC4619298; doi:10.1371/journal.pone.0138779)
Supplement: S1 File — (DOCX) [file pone.0138779.s001.docx]

**S1 File**

**Description of a new Galapagos Giant Tortoise Species (*Chelonoidis*; Testudines: Testudinidae) from Cerro Fatal on Santa Cruz Island**

Nikos Poulakakis, Danielle L. Edwards, Ylenia Chiari, Ryan C. Garrick, Michael A. Russello, Edgar Benavides, , Gregory J. Watkins-Colwell, Scott Glaberman, Washington Tapia, James P. Gibbs, , Linda J. Cayot, Adalgisa Caccone

**Materials and Methods**

All DNA extractions were performed from approximately 0.1-0.2g of museum material (bone or skin) following the protocol described by Rohland and Hofreiter [[1](#_ENREF_1)] based on the DNA IQ System (Promega, USA). Homogenized bone powder was obtained by grinding the samples using a Spex 6750 freezer mill (Spex SamplePrep, Metuchen, NJ, USA). The extractions were carried out in two physically isolated laboratories dedicated to the extraction of ancient DNA (aDNA) at Yale University and University of Crete, following all standard precautions to prevent contamination by extant specimens.

In particular, 0.2g of bone powder were incubated in 3ml digestion buffer composed of 10mM Tris (pH 8.0), 10mM NaCl, 50mM EDTA (pH 8.0), 0.5% SDS and 1mg/ml proteinase K at 56°C (overnight) under agitation. 2ml Lysis buffer (including 10mM DTT) and 15μl of Resin (magnetic particles) were added to the supernatant. After incubation at room temperature for 1h with frequent mixing of the solution, the Resin was collected using the Magnetic Stand. The supernatant was removed and again 100μl Lysis buffer were added, vortexed, the Resin separated, the solution removed and 100μl Wash buffer added. After vortexing, separation and removal of the liquid, this washing step was repeated twice. The Resin was then air dried for 30min in the Magnetic Stand and 50μl 1xTE were added, vortexed and DNA was eluted for 5min at 65°C. A negative control was used for each extraction to detect contamination throughout extraction procedures.

Approximately 700 base pairs of DNA sequence from the mitochondrial control region (CR) was amplified and sequenced in small overlapping fragments varying between 174 and 257 bp in size (including the primers) [[2](#_ENREF_2), [3](#_ENREF_3)].

For each DNA extract, PCR amplification was initially performed with a corresponding pair of external primers. The product of the primary reaction served as the template for another PCR amplification (nested reaction) using the corresponding pair of internal primers. Mitochondrial fragments were amplified in 20μL PCR reactions containing: 1X High Fidelity PCR Buffer, 3.00mM MgSO_4_, 100μM of each dNTP, 0.30μM of each PCR primer, 1mg/ml BSA and 0.4 units of Taq polymerase (Platinum Taq High Fidelity; Invitrogen). Reactions were carried out using an initial step at 94^o^C/5 min, followed by 40 cycles of 94^o^C for 45s, 50^o^C (CR), 48^o^C (cyt *b*), and 50^o^C (16S) for 60s, and 68^o^C for 90s, and a final extension of 68^o^C for 25 min. At least two sterile negative controls were used for each reaction to detect contamination throughout amplification. Double-stranded PCR products were sequenced using Big Dye 3.1 terminators on an ABI3730 DNA sequencer (Applied Biosystems, USA).

Two independent DNA extractions were performed from each bone and scale sample. Sequences were obtained from at least two amplifications of individual samples.

**References**

1. Rohland N, Hofreiter M. Comparison and optimization of ancient DNA extraction. Biotechniques. 2007;42:343-52.

2. Poulakakis N, Glaberman S, Russello M, Beheregaray LB, Ciofi C, Powell JR, et al. Historical DNA analysis reveals living descendants of an extinct species of Galápagos tortoise. Proc Natl Acad Sci USA. 2008;105(40):15464-9. doi: 10.1073/pnas.0805340105.

3. Poulakakis N, Russello M, Geist D, Caccone A. Unravelling the peculiarities of island life: vicariance, dispersal and the diversification of the extinct and extant giant Galápagos tortoises. Mol Ecol. 2012;21(1):160-73. doi: DOI 10.1111/j.1365-294X.2011.05370.x. PubMed PMID: ISI:000298582700013.
